# Phenotypes, bedside examination, and video head impulse test in vestibular migraine of childhood compared with probable vestibular migraine and recurrent vertigo in childhood

**DOI:** 10.3389/fped.2023.1152928

**Published:** 2023-06-12

**Authors:** Roberto Teggi, Bruno Colombo, Marco Familiari, Iacopo Cangiano, Mario Bussi, Massimo Filippi

**Affiliations:** ^1^ ENT Division, San Raffaele Scientific Institute, Vita e Salute University, Milano, Italy; ^2^ Units of Neurology and Neurophysiology, IRCCS San Raffaele Scientific Institute, Milan, Italy; ^3^ Neuroimaging Research Unit, INSPE, Division of Neuroscience, IRCCS San Raffaele Scientific Institute, Milan, Italy; ^4^ “Vita-Salute” University, San Raffaele, Milan, Italy

**Keywords:** vertigo, migraine, vestibular migraine in childhood, recurrent vertigo in childhood, vestibular disorders

## Abstract

**Introduction:**

Vestibular impairment and vertigo in the pediatric population have an estimated prevalence ranging between 0.4% and 5.6% and are a topic of interest in recent years. The Bárány Society has recently reclassified migraine-related vertigo syndromes as vestibular migraine of childhood (VMC), probable vestibular migraine of childhood (probable VMC), and recurrent vertigo of childhood (RVC).

**Methods:**

Applying the criteria established by the Bárány Society, we retrospectively analyzed data on 95 pediatric patients suffering from episodic vertigo that were recruited from 2018 to 2022. In applying the revised criteria, 28 patients had VMC, 38 had probable VMC, and 29 had RVC.

**Results:**

Visuo-vestibular symptoms (external vertigo) or internal vertigo were reported by 20 of 28 VMC patients (71.4%) compared to 8 of 38 probable VMC patients (21%) (*P *< .001). None of the RVC patients reported external vertigo. Duration of vertigo was demonstrably longer in the VMC patients than in the probable VMC (*P *< .001) and RVC (*P *< .001) patients. Cochlear symptoms were reported by 28.6% of VMC patients and by 13.1% of probable VMC patients. No cochlear symptoms were reported by any RVC patients. Familial cases for headache and episodic vertigo showed no significant difference between groups.

**Discussion:**

The most frequent finding during bedside examination in all three groups was central positional nystagmus. Differences in the duration of attacks and in accompanying symptoms may underline different pathophysiological mechanisms.

## Introduction

The number of publications on vestibular impairment, vertigo, and dizziness in the pediatric population has increased in the last few decades; estimates of the prevalence of vestibular disorders in childhood range widely between 0.4% and 5.6% (1–3). The limited ability of these patients to describe their symptoms and the difficulty of assessing vestibular impairment in these patients may contribute to these heterogeneous results. The association between vertigo in children and migraine is well known, since vertigo is an age-dependent symptom that is thought to represent early life expression of migraine headache. The term “benign paroxysmal vertigo of childhood” was proposed in the late “60s” to describe a clinical condition characterized by episodic vertigo, which may be associated with vomiting, pallor, and postural imbalance (4–6). This condition has been commonly accepted to be a migraine precursor (7).

The Bárány Society has recently reclassified migraine-related vertigo syndromes as vestibular migraine of childhood (VMC), probable vestibular migraine of childhood (probable VMC), and recurrent vertigo in childhood (RVC) (8). Diagnostic criteria are listed in Table 1. The authors note that RVC may be a heterogeneous condition, which, in some cases, occurs with migraine features, “but presents not (yet) enough features to fulfill the criteria for VMC or probable VMC”. The purpose of this work was to assess the clinical features, bedside examination, and familial history of migraine and vertigo in a population of VMC patients and compare the results with probable VMC and RVC patients.

**Table 1:** The Bárány Society diagnostic criteria for VMC, probable VMC and RVC.

**Vestibular migraine of childhood (VMC)**
A. At least five episodes with vestibular symptoms of moderate or severe intensity 1,2, lasting between five minutes and 72 h
B. A current or past history of migraine with or without aura
C. At least half of episodes are associated with at least one of the following three migraine features
1. Headache with at least two of the following four characteristics:
(a) One sided location
(b) Pulsating quality
(c) Moderate or severe pain intensity
(d) Aggravation by routine physical activity
2. Photophobia and phonophobia
3. Visual aura
D. Age <18 years
E. Not better accounted for by another headache disorder, vestibular disorder, or other condition
**Probable vestibular migraine of childhood (probable VMC)**
A. At least three episodes with vestibular symptoms of moderate or severe intensity, lasting between five minutes and 72 h
B. Only one of the criteria B and C for Vestibular Migraine of Childhood
C. Age <18 years
D. Not better accounted for by another headache disorder, vestibular disorder, or other condition
**Recurrent vertigo of childhood (RVC)**
A. At least three episodes with vestibular symptoms of moderate or severe intensity, lasting between 1 min and 72 h
B. None of the criteria B and C for Vestibular Migraine of Childhood
C. Age <18 years
D. Not better accounted for by another headache disorder, vestibular disorder, or other condition

## Materials and methods

### Study cohort

In this retrospective study we included 95 pediatric patients suffering from episodic vertigo recruited from 2018 to 2022. Final diagnoses were made retrospectively according to the criteria of the Bárány Society (8); 28 patients fulfilled criteria for VMC, 38 for probable VMC, and 29 for RVC. Patients were included if complete clinical data and bedside examination findings were available and retrieved from our clinical records and if an MRI of the central nervous system was negative. All patients were evaluated by a senior neurologist to assess the presence of migrainous headache. Complete clinical history was collected by a senior neurotologist, which included:

- Demographic data, age of onset of the first vertigo, duration, and features of episodes. Since it is often difficult for pediatric patients to describe vertigo, we only collected the data if the subject reported visuo-vestibular symptoms (external vertigo) or internal vertigo (false sensation of self-motion), defined as the false sensation that the visual surroundings are spinning or flowing according to the Bárány Society criteria, in more than 50% of the episodes (9). Since in our experience it was difficult to differentiate external/internal vertigo in pediatric patients, they were considered together, while other mild conditions like dizziness were excluded since they are more difficult to assess in pediatric subjects.
- Duration of vertigo attacks; patients were asked about the most frequent duration of attacks.
- Concomitant symptoms (nausea and vomiting, cochlear symptoms, photo-phonophobia, and headache) present in more than 50% of episodes.
- Duration of most vertigo attacks. Duration data were grouped as: 5–30 min [to avoid including benign paroxysmal positional vertigo (BPPV) or vestibular paroxysmia], 30 min–4 h, 4–12 h (the 4 h cutoff was chosen in light of a recent report that the duration of vertigo in patients with RVC had a range of 1 min–4 h 10), and >12 h.
- Presence of motion sickness
- Familial history of episodic vertigo and migrainous headaches (parents were asked if another first or second-degree relative suffered from migraine and/or vertigo).

All patients underwent audiometric exam confirming normal hearing. Data were stored in a protected database.

### Bedside examination

Nystagmus was studied with video Frenzel goggles (Interacoustics, Assens, Denmark).

All subjects underwent bedside examination. The following tests were performed:

- Head-shaking test (HST) with the patient sitting in a clinical chair with the head tilted downwards by 30°. The patient's head was vigorously rotated for 20 times on the horizontal plane with a maximum amplitude of 30–40°. Post HST nystagmus was recorded for 1 min and was considered positive when nystagmus lasting at least 5 s was detected.
- Skull vibration test (skull vibration induced nystagmus—SVIN test) was performed at 100 Hz with a commercially available system (VVIB—Synapsis). Stimuli were applied perpendicularly to the skin over the mastoid process, posteriorly to the auricle, at the level of the external acoustic meatus and on the midline with a force around 1 kg; three stimulation trials were performed on each mastoid, lasting 5–10 s each. Eye movements were studied with video Frenzel goggles and visual fixation of both eyes was inhibited. The test was considered positive when a horizontal nystagmus, always beating on the same side, was elicited in all 6 trials.
- Positional nystagmus was assessed in the supine position with the head tilted to both sides to an angle of 90°.
- Dix-Hallpike and Pagnini-McClure maneuvers were performed to exclude BPPV.
- Romberg and Unterberger test was performed as the final test.

Video head impulse test (video-HIT) was performed on the horizontal plane with a commercially available system (ICS Impulse, Otometrics, Taastrup, Denmark). The calibration was also checked on the horizontal canal plane since it is often difficult to perform vertical canals and there is limited examination time available for obtaining the collaboration of younger subjects. Particular attention was paid to achieving a sufficient fit of the goggles. Trials with blinks and outliers were automatically excluded. Recordings showing that eye movements preceded head movements, even after attempts to improve goggle fit, were not included in subsequent analysis.

Failure to acquire data for any of tests listed above was considered as an exclusion criterion. We considered the evaluation to be normal if the patient presented a normal gain at video-HIT without compensatory saccades, nystagmus was not elicited by various maneuvers, and the patient did not present a rotation of more than 45° in the Unterberger test. The study was approved by our internal Ethics Committee as a part of a wider study on vestibular migraine (105/INT/2014).

### Statistical analyses

Quantitative variables are presented as mean ± standard deviation, and categorical variables are presented as a rate on the total sample. *P *< .05 was considered statistically significant. Chi-square and odds ratios were calculated to establish the different frequencies of clinical signs in subjects with and without a comorbidity for migraine. A Spearman test was used to correlate age of onset of first vertigo and age of first headache in VMC patients. We used SPSS software version 22.0 (SPSS, Inc., Chicago, IL, USA) for statistical analyses.

## Results

The cohort comprised 28 VMC patients, 24 (85.7%) of whom were females, 38 probable VMC patients, 23 (60.5%) of whom were females, and 29 RVC patients 15 (51.7%) of whom were females. The proportion of females was significantly higher in the VMC group compared to the other two groups (*χ*^2^* *= 7.8867, *P *= .02).

### Clinical data

The age of onset of the first vertigo was 15.6 ± 1.8 years (range 11–17) in the VMC group, 12.3 ± 1.9 years (range 9–17) in the probable VMC group, and 8.6 ± 2.3 years (range 5–12) in the RVC group. The age of onset of the first migrainous headache was 14.3 ± 1.7 years (range 10–16) in the VMC group, correlating with the age of onset of the first vertigo (*t* = 5.05, *P *< .001; Spearman test). In the probable VMC group, the age of onset of the first migrainous headache in a subset of 18 patients between 13 and 17 years was 13.9 ± 2.1 years (range 13–17), also correlating with the age of onset of first vertigo (*t* = 2.46, *P *= .02).

The most frequent duration of attacks of vertigo are reported in Table 2. Longer duration was seen in the VMC group compared with the probable VMC group (*χ*^2 ^= 24, *P *< .001) and the RVC group (*χ*^2 ^= 40, *P *< .001).

**Table 2:** Duration of vertigo attacks in the group of VMC, probable VMC and RVC.

	VMC	Probable VMC	RVC	
Less 30 min	3/28 (10.8%)	15/38 (39.5%)	20/29	(68.9%)
30 min to 4 h	14/28 (50%)	15/38 (39.5%)	8/29 (27.6%)	
4–12 h	5/28 (17.8%)	5/38 (13.6%)	0/29	
Over 12 h	6/28 (21.4%)	3/38 (7.9%)	1/29	(3.5%)

Between parentheses. A longer duration of attacks has been found in the VMC compared to probable VMC* (*P *< .001) and RVC* (*P *< .001).

The occurrence of external vertigo (visuo-vestibular symptoms) or internal vertigo (false sensation of self-motion) in most attacks were reported by 20 of 28 patients (71.4%) in the VMC group and by 8 of 38 patients (21%, *χ*^2 ^= 16.7, *P *< .001) in the probable VMC group; none of the patients in the RVC group reported external or internal vertigo in most attacks.

Among concomitant symptoms, nausea and vomiting were reported in most attacks by 25 of 28 patients (89.3%) with VMC; among these, 21 of 28 (75%) reported migrainous headache during vertigo. Among probable VMC, 26 of 38 patients (68.4%, *P *= .08) reported nausea and vomiting, and among RVC, 20 of 29 patients (68.9%, *P *= .059) reported nausea and vomiting.

Cochlear symptoms were reported by 8 of 28 (28.6%) of patients with VMC, with 5 reporting tinnitus and 3 fullness; cochlear symptoms were reported by 5 of 38 patients (13.1%) with probable VMC, with 3 reporting tinnitus and 3 fullness (*P *= .12). No cochlear symptoms were reported by patients in the RVC group.

No hearing loss was reported by subjects in any of the three groups, and all patients had normal hearing. No significant difference was observed for motion sickness, which was reported by 23 of 28 (82.1%) patients with VMC, 25 of 38 (66%) with probable VMC (*P *= .14), and 18 of 29 with RVC (*P *= .09).

Familial cases for headache and episodic vertigo were investigated in parents and grandparents. Vertigo was reported by parents/grandparents of 8 of 28 patients (28.6%) with VMC, by parents/grandparents of 5 of 38 (13.1%) patients with probable VMC (*χ*^2 ^= 2.4, *P *= .12), and by parents/grandparents of 3 of 29 patients (10.3%) with RVC (*χ*^2 ^= 3, *P *= .08). Familial cases of headache with migrainous features were reported by by parents/grandparents of 20 of 28 patients (71.4%) with VMC, by parents/grandparents of 27 of 38 patients (71%) with probable VMC (*P *= .97) and by parents/grandparents of 12 of 29 patients (41.4%) with RVC (*t* = 5.2, *P *= .02).

### Bedside examination

A positive SVIN test was found in one patient with VMC and in one patient with probable VMC; no patient in any group presented a positive video-HIT, a post head shaking test nystagmus, or positive tests for BPPV during examination. Bi-positional apogeotropic nystagmus of low frequency, without vertigo, normally without latency, and long lasting (more than 5 min of observation) was seen in all three groups. This finding was present in 6 of 28 patients (21.4%) with VMC, 7 of 38 patients (18.4%) with probable VMC (*χ*^2^* *= 0.1, *P *= .76), and 2 of 29 (6.9%) patients with RVC (*χ*^2^* *= 2.4, *P *= .11).

Clinical data and statistics are summarized in Tables 3 and 4.

**Table 3:** Clinical features of vertigo, accompanying symptoms and family history for vertigo and migraine in the sample of probable VMC and VMC.

	Probable VMC	VMC	*P*
Sex—female	23/38 (60.5%)	24/28 (85.7%)	.02
Age of onset of vertigo (year)	12.3 ± 1.9	15.6 ± 1.8	.002
Vertigo (%)	8/38 (21%)	20/28 (71.4%)	<.001
Nausea (%)	26/38 (68.4%)	25/28 (89.3%)	.08
Cochlear symptoms (%)	5/38 (13.1%)	8/28 (28.6%)	.12
Motion sickness (%)	25/38 (66%)	23/28 (82.1)	.14
Familial vertigo (%)	5/38 (13.1%)	8/28 (28.6%)	.12
Familial headache (%)	27/38 (71%)	20/28 (71.4%)	.97
Positional nystagmus (%)	7/38 (18.4%)	6/28 (21.4%)	.76

Total number (and percentage) are reported. As for vertigo, nausea and cochlear symptoms are reported the total number of patients with symptoms present in most attacks (more than 50%).

**Table 4:** Clinical features of vertigo, accompanying symptoms and familiarity for vertigo and migraine in the sample of VMC and RVC.

	RVC (*N* = 29)		VMC (*N* = 28)	*P*
Sex—female	15/29	(51.7%)	24/28 (85.7%)	.01
Age of onset of vertigo (year)	8.6 ± 2.3	15.6 ± 1.8	<.001	
Vertigo (%)		0	20/28 (71.4%)	
Nausea (%)	20/29	(68.9%)	25/28 (89.3%)	.059
Cochlear symptoms (%)		0	8/28 (28.6%)	
Motion sickness (%)	18/29 (62%)	23/28 (82.1)	.09	
Familial vertigo (%)	3/29 (10.3%)	8/28 (28.6%)	.08	
Familial headache (%)	12/29	(41.4%)	20/28 (71.4%)	.02
Positional nystagmus (%)	2/29	(6.9%)	6/28 (21.4%)	.11

Total number (and percentage) are reported. As for vertigo, nausea and cochlear symptoms are reported the total number of patients with symptoms present in most attacks (more than 50%).

**Figure 1:**
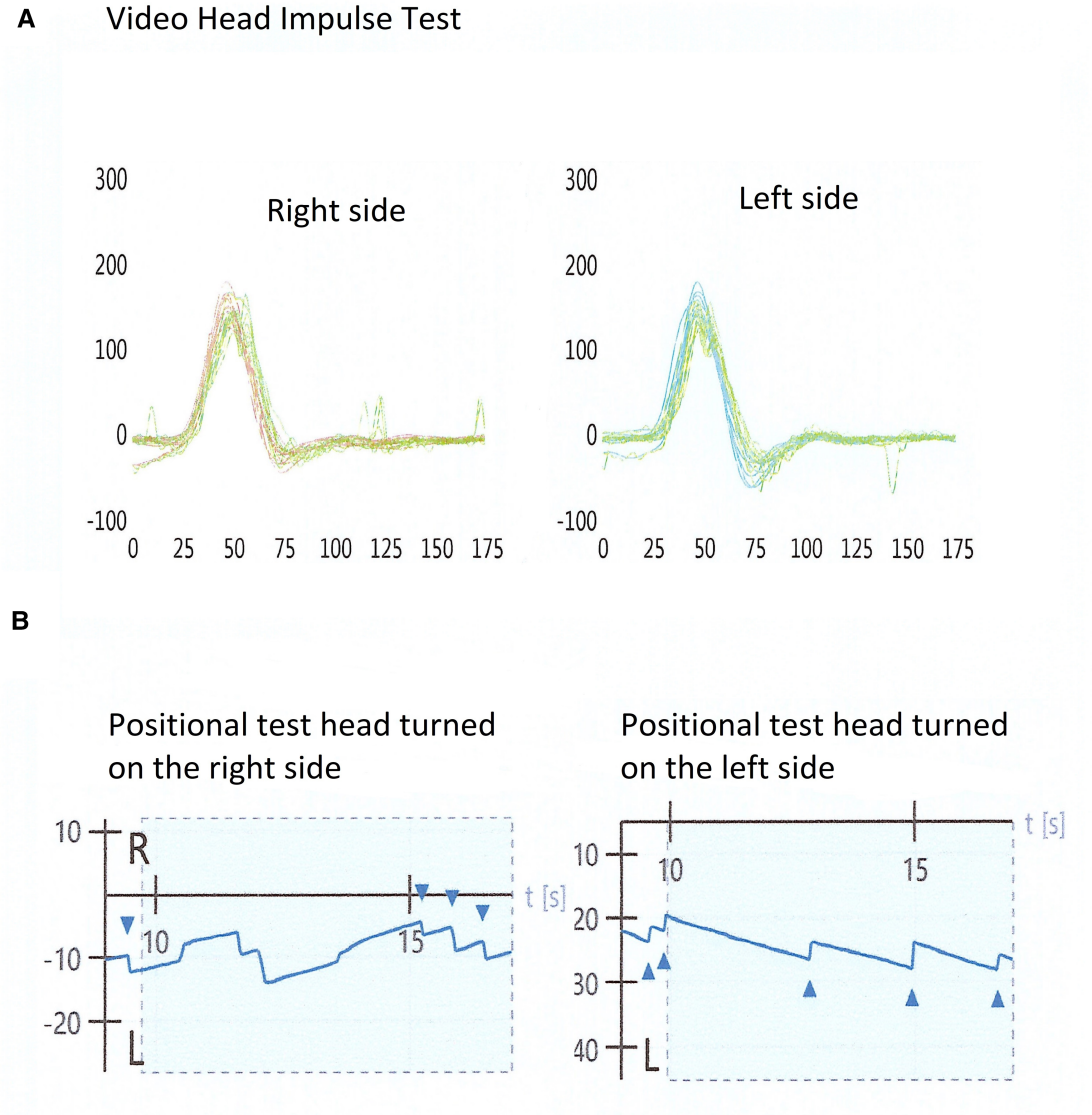
Examination of a patient with positive bi-positional apogeotropic nystagmus and a normal video head impulse test. (**A**) A normal video head impulse test on the horizontal plane; (**B**) a bi-positional apogeotropic nystagmus, with head turned on the right and left side.

In Figure 1 a typical exam with a normal video head impulse and bipositional apogeotropic nystagmus is shown.

## Discussion

Benign paroxysmal vertigo has long been considered to be the most frequent disorder provoking vertigo in pediatric patients, and there is a general consensus that vertigo shares pathophysiological mechanisms with migraine in pediatric patients. Benign paroxysmal vertigo is characterized by recurrent attacks of vertigo associated with nausea/vomiting, pallor, and postural imbalance in otherwise healthy subjects and is considered to be a migraine precursor by several authors (11–14). In the original work of Basser, the age of onset of the condition was reported to be around 4 years of age, although the condition is not limited to childhood (7). Benign paroxysmal vertigo has been included in the International Classification of Headache Disorders as a migraine precursor (15).

To date, few studies have examined the phenotypes of vertigo in VMC, probable VMC, and RVC. Although our cohort is relatively small, in our opinion it nonetheless provides some interesting information. In our sample of VMC and probable VMC, females were more prevalent, a finding in line with previous studies reporting a similar percentage in adults with vestibular migraine. It could be hypothesized that these subjects present a profile similar to VM in adults (5).

The age of onset of vertigo in our sample of VMC was 15.6 years (range 11–17), which is later than onset of probable VMC and RVC, although another study reported that the age of onset was 10.7 years in a sample of 22 subjects (16). Our data are not different from those of other investigations reporting the onset of vertigo in a sample of subjects around 14 years in which comorbidity for migraine was not assessed (17). In our study, the onset of headaches preceded vertigo by 18 months, which is lower than in VM in adults. It is possible that reported differences in the onset of vertigo may be linked to the selection criteria in different studies as well as to the limited number of patients. It is also possible that a proportion of the probable subjects can evolve towards a “definite” VMC disorder.

VMC patients in our study reported a longer duration of vertigo compared with other 2 samples, lasting more than 12 h in 21.4% of the cases, and a higher rate of visuo-vestibular symptoms (external vertigo, i.e., the sensation that the surrounding are flowing) in 71.4% of cases. These findings resemble attacks of definite VM in adults (18, 19). Our results can be only compared with those of a sample of pediatric subjects, reporting vertigo in over 50% of cases (20, 21). Among accompanying symptoms, nausea was a common complaint in our sample among all three groups, ranging between 68.9% and 89.3%. Cochlear symptoms were reported by 28.6% of VMC patients and 13.1% of probable VMC patients but were not reported by any patient in the RVC group. This frequency is lower than those of another recent study, which reported hearing complaints in 42% of patients with episodic vertigo (20).

Video head impulse and vibration-induced nystagmus were negative in almost all subjects; in a vertigo free period, bi-positional direction changing nystagmus, mostly apogeotropic, was the main finding in both the probable VMC and VMC groups. These results are in line with those of VM in adults and are considered as a sign of a central vestibular disorder. Central positional nystagmus is the most common finding in adult VM, possibly arising from the disruption of brainstem or cerebellar vestibular networks (22–24).

The pathophysiology of VM is still under debate, and both central and peripheral mechanisms have been proposed. Migrainous headache is thought to result from increased activity or sensitization within the trigeminovascular system. Neural projections to vestibular nuclei might affect the sensitivity of vestibular nuclei resulting in migraine-associated vestibular dysfunction (25). On the other hand, a vasospasm of the internal auditory artery may be postulated, since the vascular circulation of the inner ear receives innervation from the trigeminal nerve (26). Our data suggest the presence of coexisting mechanisms, one related to bedside findings of a central vestibular disorder and a second related to the concomitant cochlear symptoms.

Finally, we found a high rate of familial cases of migraine (around 80%) in both probable VMC and VMC, while 28.6% of VMC subjects had familial cases of episodic vertigo. These findings overlap with those of previous studies in adult VM and in benign paroxysmal vertigo in childhood. and higher than in samples of migraineurs without vertigo. These findings are not inconsistent with the possibility of different genetic mechanisms related to migraine *per se* and VM (19, 27).

Our study has potential limitations. According to the criteria of the Bárány Society, diagnosis relies exclusively on clinical history. This represents a limitation arising from the difficulty of obtaining a careful medical history in children. Moreover, during clinical vestibular tests, good compliance is not always possible with children. Another potential limitation is the lack of follow-up over time, We cannot exclude the possibility that a form previously characterized as RVC could shift into VMC over time. Only horizontal canal was performed during video HIT in order to limit the time of collaboration of the patient, and it cannot be excluded that we missed a vertical canal deficit because of this choice. Moreover, VEMPs were not performed, and macular disorders in these subjects cannot be excluded. Finally, we decided to include only patients reporting external/internal vertigo, (i.e., the sensation that the surrounding is flowing) to avoid conditions, like dizziness, in which emotional factors may play an important role. Because of this choice, we are aware that patients fulfilling the criteria for VMC may have been excluded. On one hand, the difficulty in collecting clinical history in pediatric subjects should be taken into account. On the other hand, it is difficult to differentiate external and internal vertigo, therefore, we considered them together as a single variable.

## Data Availability

The raw data supporting the conclusions of this article will be made available by the authors, without undue reservation.
